# Glomerular cell atlas of multi-disease model revealed the characteristic changes of glomerular cell subtypes in diseases

**DOI:** 10.3389/fimmu.2026.1763345

**Published:** 2026-02-24

**Authors:** Yan Huang, Shuo Li, Shuying Li, Shuzhong Duan, Lan Huang, Jing Wang, Liangyan Ma, Ce Liu, Qilin Chen

**Affiliations:** 1 Nephrology Department, The Affiliated Hospital of Chengde Medical University, Chengde, China; 2 Metabolic Vascular Disease Group, Hebei Key Laboratory of Panvascular Diseases, Chengde, China; 3 Department of Nephrology Children’s Hospital of Chongqing Medical University, National Clinical Research Center for Children and Adolescents’ Health and Diseases, Ministry of Education Key Laboratory of Child Development and Disorders, Chongqing Key Laboratory of Pediatric Metabolism and Inflammatory Disease, Chongqing, China

**Keywords:** cross-disease atlas, glomerular disease, mesangial cell, podocyte, single-cell RNA sequencing

## Abstract

Although a range of glomerular diseases profoundly affect glomerulus-associated cells, a comprehensive understanding of their molecular alterations is still lacking. Here, we performed in-depth analysis of glomerular data from mouse models of primary and secondary glomerulopathies and constructed a multi-disease cellular landscape of glomerular cells. We identified a putative subset of proliferative glomerular endothelial cells(gECs) that highly expresses genetic susceptibility genes associated with multiple glomerular diseases. Podocytes exhibited shared injury-associated cell types across different disease models. A podocyte subset highly expressing *Endou*, *Cd200*, *Lgmn*, *Il18*, *Dmpk*, and *Spon2* was predominantly derived from ob/ob mice, whereas another podocyte subset with high expression of *Selenbp1*, *Lpar1*, *S100a8*, *S100a9*, and *Sult1a1* was mainly observed in adriamycin-induced mice. Mesangial cells shared common injury-related alterations across diseases (high expression of *Cxcl1*, *egr1*, *hspa1b*, *socs3* and *dnajb1*), while ob/ob mice exhibited a distinct mesangial cell subset (high expression of *aldh1a2, thbs1* and *fbln5*). In contrast, the gECs displayed similar molecular changes across different diseases without giving rise to disease-specific subtypes. Intercellular ligand-receptor analysis underpins the recruitment of immune cells by injured mesangial cells and podocytes via specific engagement of pairs such as CXCL and MIF, respectively. Our study systematically elucidates the molecular alterations of glomerulus-associated cells across various diseases, providing a foundation and strategic insights for future targeted therapies tailored to specific glomerular disease contexts.

## 1 Introduction

The kidney is an extremely important organ in the human body, playing a crucial role in maintaining the stability of the internal environment and normal metabolic processes. The glomerulus is a highly specialized filtration unit composed of capillary tufts, the Bowman capsule, and three layers of filtration membranes. This intricate structure enables the efficient separation of plasma water and small solutes from the blood, thereby forming the primitive urine. Furthermore, the glomerulus contributes to regulating systemic blood pressure and maintaining fluid-electrolyte homeostasis through some proteins, including renin (1). As the basic functional unit of the kidney, it is mainly composed of terminally differentiated epithelial cells—podocytes, which cover the pericytes of the glomerular capillaries—mesangial cells (MCs), glomerular endothelial cells (gECs), and other less common cell types. These cells collectively undertake a variety of different functions. Both primary glomerular diseases, which are confined to the glomeruli, and secondary glomerular diseases, resulting from systemic disorders, can cause damage to function and architecture of glomerular cells, leading to hematuria, proteinuria, edema, and decreased glomerular filtration rate (2, 3).

In podocytopathies characterized by proteinuria or nephrotic syndrome, foot process effacement (FPE) and foot process simplification represent the earliest morphological manifestations of podocyte injury. Notably, substantial proteinuria may manifest even in the absence of podocyte injury (4). The reorganization of the actin cytoskeleton is pivotal in the pathogenesis of FPE (4). Dysregulation among key actin cytoskeleton regulators can directly induce FPF, particularly involving the Rho family of small GTPases, including RhoA, CDC42, and RAC1 (5). In diabetic nephropathy, the diabetic milieu induces “pathological-adaptive” alterations in podocytes, characterized by cytoskeletal remodeling, dedifferentiation, apoptosis, and autophagy (6, 7). Simultaneously, mesangial cells sustain significant diabetic injury, showing proliferation, hypertrophy, and concomitant upregulation of extracellular matrix protein production (8). Impairments in the endothelial cells caused by toxins, antibodies, immune cells, or inflammatory cytokines, or deficiencies in endothelial protective factor (such as regulators of complement or angiogenesis), can lead to either acute or chronic kidney injury (9). Endothelial-to-mesenchymal transition contributes to the pathogenesis of renal fibrosis and the progression of chronic kidney disease by promoting the phenotypic transformation of endothelial cells toward mesenchymal phenotype (10).

Although glomerular cells are essential in maintaining normal kidney function and in the development of related diseases, there is still a lack of clear research and systematic description of the characterization changes of mouse glomerular cells under different pathological and physiological conditions. Research in this area urgently requires in-depth exploration to uncover the functional characteristics of glomerular cells under various conditions and their potential roles in kidney diseases, thereby offering more precise targets and strategies for clinical treatment. In recent years, single-cell RNA sequencing (scRNA-seq) technology has fundamentally enhanced our ability to characterize glomerular cells (11).

This study used scRNA-seq to comprehensively characterize the glomerular cell types in healthy mice and four different disease models, each mimicking a renal disease: the nephritis model (lupus nephritis or Goodpasture disease), the adriamycin-induced model (focal segmental glomerulosclerosis), the CD2AP knockout model (sporadic nephrotic syndrome or focal segmental glomerulosclerosis), and the diabetic model (diabetic nephropathy, DN). It successfully depicted the comprehensive transcriptional profiles of all cell types in the glomeruli. This work not only offers a thorough and integrated view at the single-cell level for elucidating the physiological and pathological characteristics of the glomeruli but also provides crucial data for understanding the characteristics of all cell types in the murine glomeruli under various pathological and physiological conditions. Through detailed analysis of the transcriptional spectra of these cells, we have uncovered the role of glomerular cells in kidney diseases, offering new insights for targeted therapies. These discoveries not only deepen our understanding of the pathogenic mechanisms of kidney diseases but also pave the way for the potential development of new treatment strategies and drug targets.

## 2 Method

### 2.1 Single-cell data and quality control

The single-cell RNA sequencing data used in this study was downloaded from the Gene Expression Omnibus (GEO) database, specifically from dataset GSE146912. Data processing was performed using Seurat version 4.4.0, which is widely used for single-cell RNA-seq analysis. Quality control (QC) measures were implemented to ensure the reliability of the data. The QC criteria included the requirement that each gene must be expressed in at least 1% of the cells, and the number of genes detected in each cell was constrained to a range of 400 to 7,000. Additionally, cells with a mitochondrial gene percentage greater than 5% and those with a high percentage of red blood cell-related genes (>1%) were excluded to minimize potential sources of contamination or low-quality data.

To further improve data quality, doublets (i.e., events where two or more cells are incorrectly captured as a single cell) were identified and removed using the DoubletFinder (version 2.0.3) algorithm. Subsequent cell clustering was based on these identified variable genes. The identification of differentially expressed genes (DEGs) was performed using the Seurat function FindAllMarkers, which helps in the detection of genes that are uniquely expressed in each cluster of cells. Cluster annotation was carried out by referencing typical cell-type-specific markers in the literature.

To address potential batch effects, which are common in large-scale single-cell datasets, the Harmony algorithm (version 1.2.0) was employed to integrate the data and mitigate these batch effects, ensuring more accurate and robust downstream analysis. This approach ensures high-quality and reliable results for subsequent analyses and interpretations.

### 2.2 Cell communication analysis

In this study, we conducted cell communication analysis using CellChat V1.6.1, with the mouse database selected for the analysis. CellChat is a powerful tool that integrates prior knowledge of ligand-receptor interactions and their cofactors to model the probability of cell communication from single-cell RNA-seq data. The analysis process involves several key steps. First, we created a CellChat object by inputting the normalized data and cell metadata. Then, we set the mouse database in the CellChat object. Next, we preprocessed the expression data to identify overexpressed genes and interactions, which are crucial for subsequent communication inference. After that, we applied the projectData function to smooth the data using protein-protein interaction (PPI) data, enhancing the reliability of communication predictions. Subsequently, we computed the communication probabilities and filtered the interactions based on a minimum cell threshold. Finally, we aggregated the communication network at the pathway level. To assess intergroup differences, we performed differential interaction analysis using the netVisual_diffInteraction function. All visualizations were generated using built-in CellChat functions.

### 2.3 Transcription factor analysis

We conducted transcription factor analysis using pySCENIC V0.12.1, a powerful Python-based tool for inferring gene regulatory networks from single-cell RNA-seq data. The analysis process involves three main steps. First, we constructed a co-expression network of transcription factors and potential target genes using the grn command with the GRNBoost2 algorithm, which identifies regulatory relationships based on gene expression correlations. Next, we refined the network by integrating motif information with the ctx command, using the mm10_10kbp_up_10kbp_down_full_tx_v10_clust.genes_vs_motifs.rankings.feather file as input. This step filters out non-significant interactions and retains only those supported by motif evidence. Finally, we assessed the activity of each regulon across all cells using the aucell command, which provides a quantitative measure of regulon activity in each cell. The visualization process followed the SCENIC (R) tutorial, ensuring a comprehensive presentation of the results.

### 2.4 Pseudotime analysis

We performed pseudotime analysis using Monocle2 V2.26.0. The analysis workflow involved several key steps. First, we identified differentially expressed genes across clusters using the differentialGeneTest function with a full model formula that included cluster information. The top 1000 genes identified from this analysis were used to filter the dataset with the setOrderingFilter function. Next, we applied dimensionality reduction using the reduceDimension function with the DDRTree method, which is the default in Monocle2 and helps visualize the data in a low-dimensional space. Finally, cells were ordered along the inferred trajectory using the orderCells function. Additionally, genes that varied along the pseudotime trajectory were also identified using differentialGeneTest, and their expression patterns were visualized with the corresponding Monocle2 functions.

### 2.5 Gene set enrichment analysis

In this study, we employed the clusterProfiler package to conduct Gene Ontology (GO) and Kyoto Encyclopedia of Genes and Genomes (KEGG) enrichment analyses. For each cluster, the top 50 differentially expressed genes were selected as input for enrichment analysis. The enrichGO function was used to categorize genes into biological process (BP), molecular function (MF), and cellular component (CC), providing insights into their functional roles. Meanwhile, the enrichKEGG function identified significantly enriched signaling pathways associated with each cluster. This approach allowed us to systematically explore the biological significance of each cluster, uncovering potential functional pathways and molecular mechanisms involved in the dataset.

### 2.6 Subcluster analysis

For subcluster identification, relevant cell types were extracted, followed by processing similar to the standard single-cell analysis workflow, except that no additional quality control (QC) was performed. The analysis was conducted using Seurat, following its standard pipeline. Specifically, NormalizeData(normalization.method = “LogNormalize”) was applied to normalize gene expression, and FindVariableFeatures(selection.method = “vst”, nfeatures = 2000) was used to identify highly variable genes. The ScaleData function was then applied to the highly variable genes identified previously, to remove unwanted sources of variation.

RunPCA(features = VariableFeatures(object)) was performed for dimensionality reduction, followed by batch effect correction using the Harmony algorithm. FindNeighbors(dims = 1:30, reduction = “harmony”) was then used to construct the nearest-neighbor graph. For clustering, FindClusters was run iteratively with the resolution parameter ranging from 0.1 to 1 to explore different clustering granularities. The optimal number of clusters was determined using the clustree package, which visualizes clustering results across different resolutions to aid in selecting an appropriate resolution value.

RunUMAP and RunTSNE were used for visualization, and marker genes were identified using FindAllMarkers with default parameters to annotate subclusters based on known cell type markers.

### 2.7 Immunofluorescence

The paraffin-embedded sections were prepared for immunofluorescence. Antigen retrieval for the paraffin-embedded sections was conducted using high-temperature pressure in Tris-EDTA buffer (pH 9.0). Tissue sections were blocked with goat serum (Biosharp, BL1097A) and incubated overnight with primary antibodies (S100a8, R&D, MAB3059; Wilms Tumor 1, Immunoway, YM6533). Fluorescent secondary antibodies (Goat Anti-Rat IgG H&L, Abcam, Cat# ab150159; Goat anti-Mouse IgG (H+L), Invitrogen, Cat#A11001) were incubated for 1 hour. Nuclear staining was performed using DAPI (Biosharp, BL120A). Images were captured using a laser scanning confocal microscope.

## 3 Results

### 3.1 Construction of mouse glomerular cell atlas and analysis of expression characteristics of pathogenic genes of kidney disease

**Figure 1:**
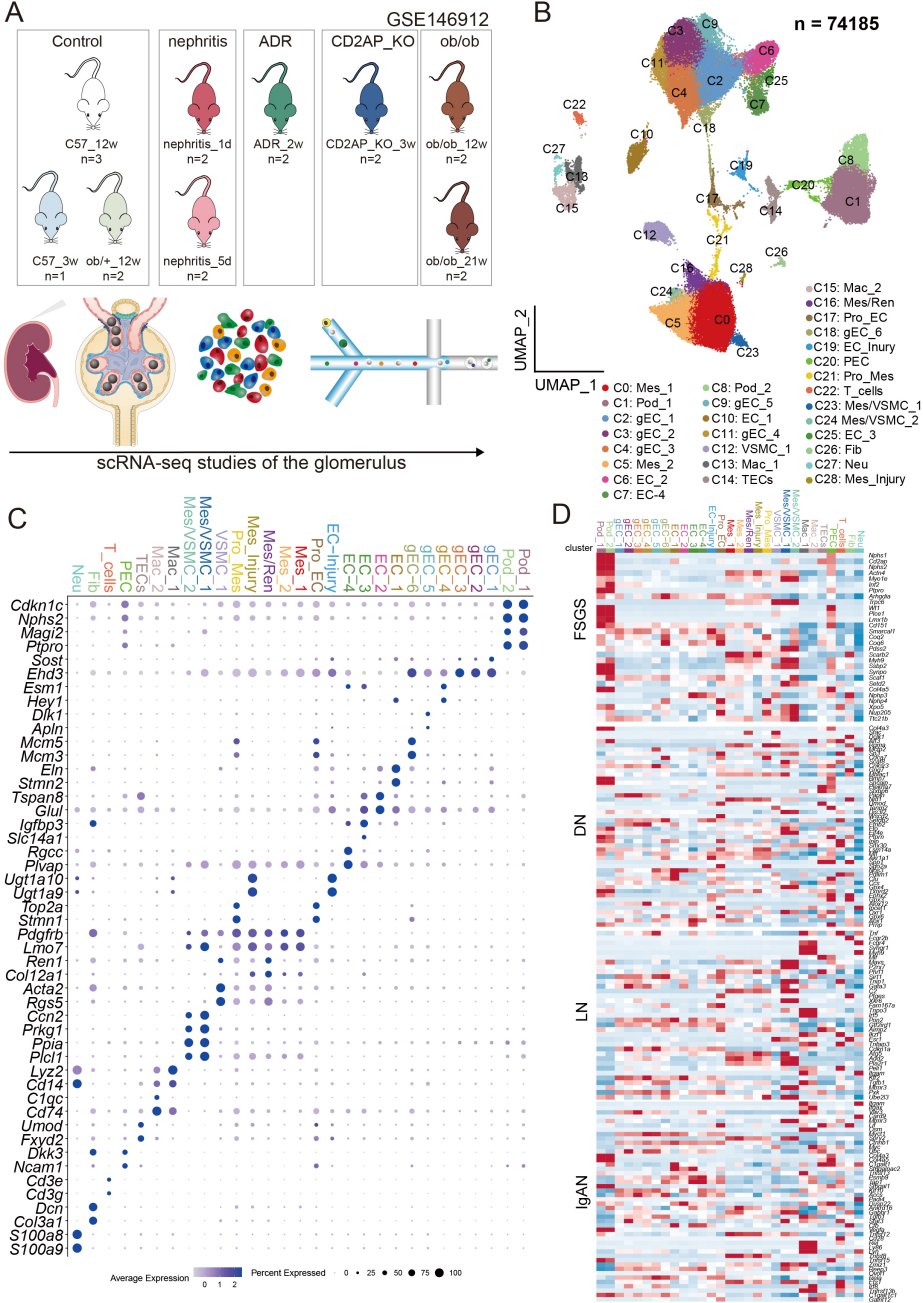
Construction of mouse glomerular cell atlas and analysis of expression characteristics of pathogenic genes of kidney disease. **(A)** The schematic diagram of this research. **(B)** UMAP of 74,185 glomerular cells. Mes, mesangial Cell; POD, podocyte; gEC, Glomerular endothelial cell; EC, endothelial cell; VSMC, vascular smooth muscle cell; Mac, macrophage; TEC, tubular epithelial cells; PEC, parietal epithelial cell; Ren, renin cell; Pro, proliferating; T, T cell; Fib, fibroblast; Neu, Neutrophil. **(C)** Dot plot of expression of cell type-specific marker genes for each glomerular cell subset. The size of each dot indicates the percentage of cells expressing the marker gene, while the color intensity reflects the average expression level of the marker gene. **(D)** Heatmap of the expression profiles of pathogenic single genes in common kidney diseases across different subgroups.

To explore the changes in specific gene expression in mouse glomerular cells under differentpathological and physiological backgrounds, we intentionally included mice of different ages (**Supplementary Table 1**), with different disease backgrounds, and at different disease durations for a comprehensive and integrated analysis of the mouse glomerulus (**Figure 1A**). We integrated the enriched single-cell transcriptomic sequencing data (GSE146912) of glomeruli and conducted strict quality control and batch effect correction (Method). Through unsupervised clustering, we obtained 29 subpopulations (**Figure 1B**). Utilizing classical kidney cell markers (**Supplementary Table 2**), we identified and characterized the kidney cell subpopulations predominantly composed of glomerular cells, including podocytes (PODs), mesangial cells (MCs), endothelial cells (gECs), and parietal epithelial cells (PECs) (**Figure 1C**). Different from other kidney single-cell omics data (11–14), this dataset had captured a certain number of glomerular-related cell subsets (15, 16), which had provided the possibility for subsequent analysis.

We systematically profiled the expression of susceptibility genes identified through genome-wide association studies (GWAS) for major kidney diseases (focal segmental glomerulosclerosis-FSGS, diabetic nephropathy-DN, lupus nephritis-LN, immunoglobulin A nephropathy-IgAN) across diverse renal cell subtypes, and delineated their cell type–specific enrichment patterns (**Figure 1D**). Genes linked to FSGS were highly expressed primarily within glomerular cells, notably PODs, PECs, and MCs, but exhibited minimal influence on gECs, extraglomerular endothelial cells, or various immune cells. Conversely, DN-associated genes were broadly expressed across diverse renal cell subtypes. For LN, the pathogenic genes were not prominently expressed in PODs but were predominantly enriched in MCs and MAC-related subsets. In contrast, IgAN-associated genes were widely active in gECs, extraglomerular endothelial cells, and immune cells, yet showed limited expression in other intrinsic glomerular cells.

Notably, we identified a high expression of multiple disease-associated molecules in Cluster 17 (pro_EC). This subpopulation expresses gEC markers (*Sost*, *Ehd3*) along with characteristic proliferation-related molecules (*Top2a*, *Stmn1*). These results suggest the existence of a distinct proliferative gEC population within the glomerulus, which may play a pivotal role in disease pathogenesis.

### 3.2 Distinct molecular signatures of podocyte injury across disease contexts

**Figure 2:**
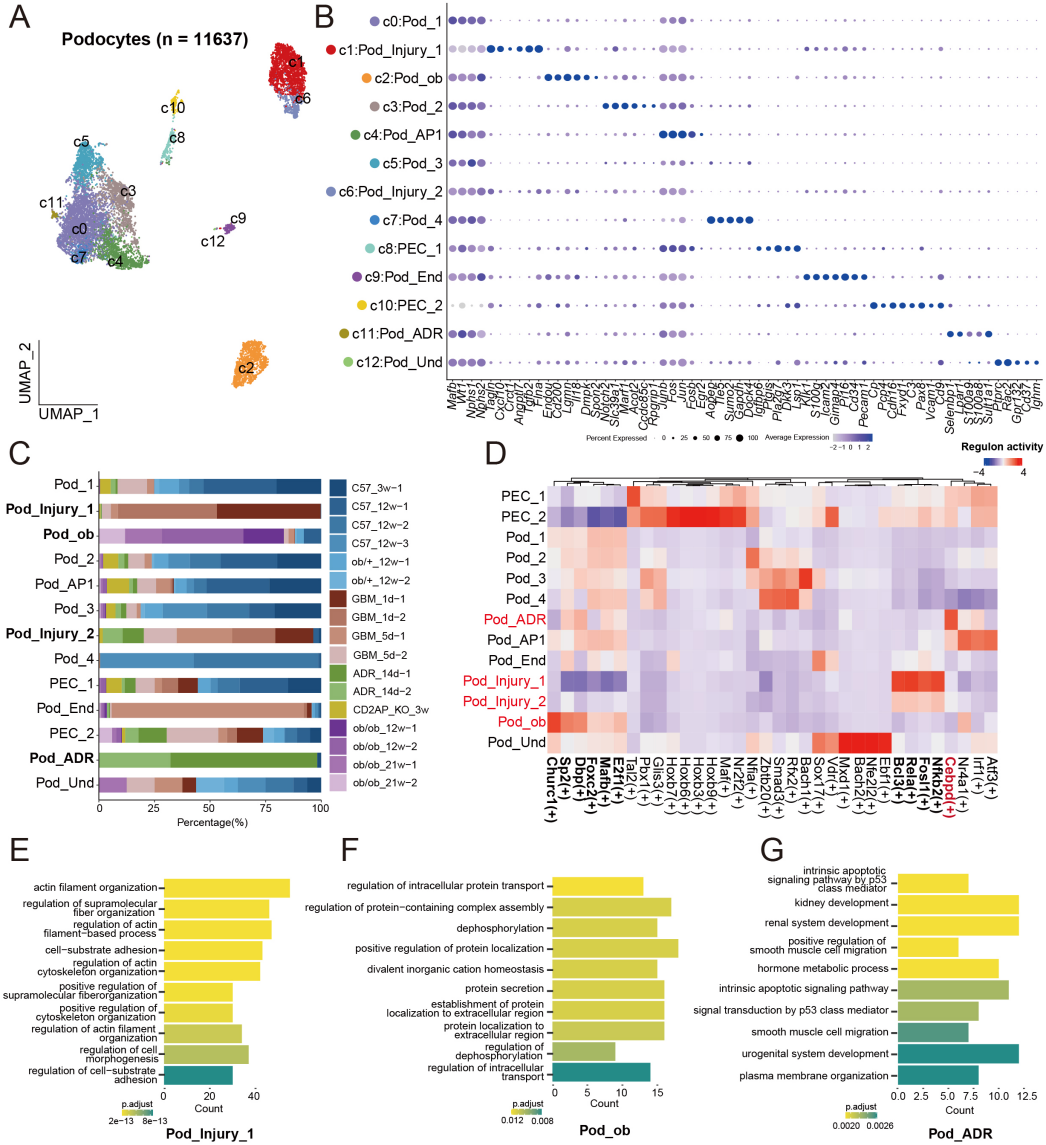
Distinct molecular signatures of podocyte injury across disease contexts. **(A)** UMAP of 11,637 podocytes. **(B)** Dot plot of expression of cell type-specific marker genes for each podocyte subset. The size of each dot indicates the percentage of cells expressing the marker gene, while the color intensity reflects the average expression level of the marker gene. **(C)** Bar chart of podocyte subset proportions across different animal models. **(D)** Heatmap of characteristic transcription factors in different podocyte subsets. **(E, F)** Bar chart of GO enrichment analysis for different podocyte subsets.

Given the critical role of POD and PEC injury in proteinuria pathogenesis (4, 17, 18), we performed an integrated re-clustering analysis of these populations, which identified 13 distinct subclusters (**Figure 2A**). Clusters 8 and 10 both expressed established PEC markers (*Igfbp6*, *Ptgis*, *Pla2g7* and *Dkk3*) (**Figure 2B**). Cluster 8 was designated as PEC_1 based on its high expression of AP-1-related molecules (*Junb*, *Jun*, *Fos* and *Fosb*). In contrast, Cluster 10, characterized by elevated expression of *Cp*, *Pcp4*, *Cdh16*, *Fxyd1*, *C3*, *Pax8*, *Vcam1*, and *Cd9*, was designated PEC_2. Notably, while PEC_1 contained cells derived from both healthy and diseased mice, the PEC_2 population was predominantly contributed by the disease group (**Figure 2C**).

Distinct injured podocyte subpopulations emerge across disease models (**Figures 2A, B**). These clusters exhibit unique molecular signatures (**Figure 2B**), disease origins (**Figure 2C**), transcription factor activities (**Figure 2D**), and functional enrichments (**Figures 2E–G**). POD_Injury_1, present across multiple disease models (**Figure 2C**), highly expresses *Tagln*, *Cxcl1*, *Crct1*, *Angptl7*, *Tgfb2*, and *Flna* (**Figure 2B**), suggesting a common injury response signature shared among different nephropathies. In contrast, Cluster 2 exhibits high expression of *Endou*, *Cd200*, *Lgmn*, *Il18*, *Dmpk*, and *Spon2* (**Figure 2B**) and originates almost exclusively from *ob/ob* mice (**Figure 2C**), prompting its annotation as POD_ob. Similarly, Cluster 11, predominantly originating from ADR-treated mice, was designated POD_ADR based on elevated expression of *Selenbp1*, *Lpar1*, *S100a8*, *S100a9*, and *Sult1a1*. Transcription factor analysis revealed that POD_ob specifically expresses *Churc1*, *Sp2*, *Dbp*, *Foxc2*, *Mafb*, and *E2f1*, while POD_Injury_1 and POD_Injury_2 both upregulate *Bcl3*, *Rela*, *Fosl1* and *Nfkb2*. POD_ADR is characterized by high expression of *Cebpd* (**Figure 2D**). GO analysis indicated that POD_Injury_1 (**Figure 2E**) and POD_ob (**Figure 2F**) are both enriched in terms related to cytoskeleton and actin filament organization. POD_ADR, however, is primarily involved in intracellular signaling, protein transport and secretion, smooth muscle cell migration, and apoptotic signaling pathways (**Figure 2G**).

### 3.3 Shared molecular signature of podocyte injury across disease contexts

**Figure 3:**
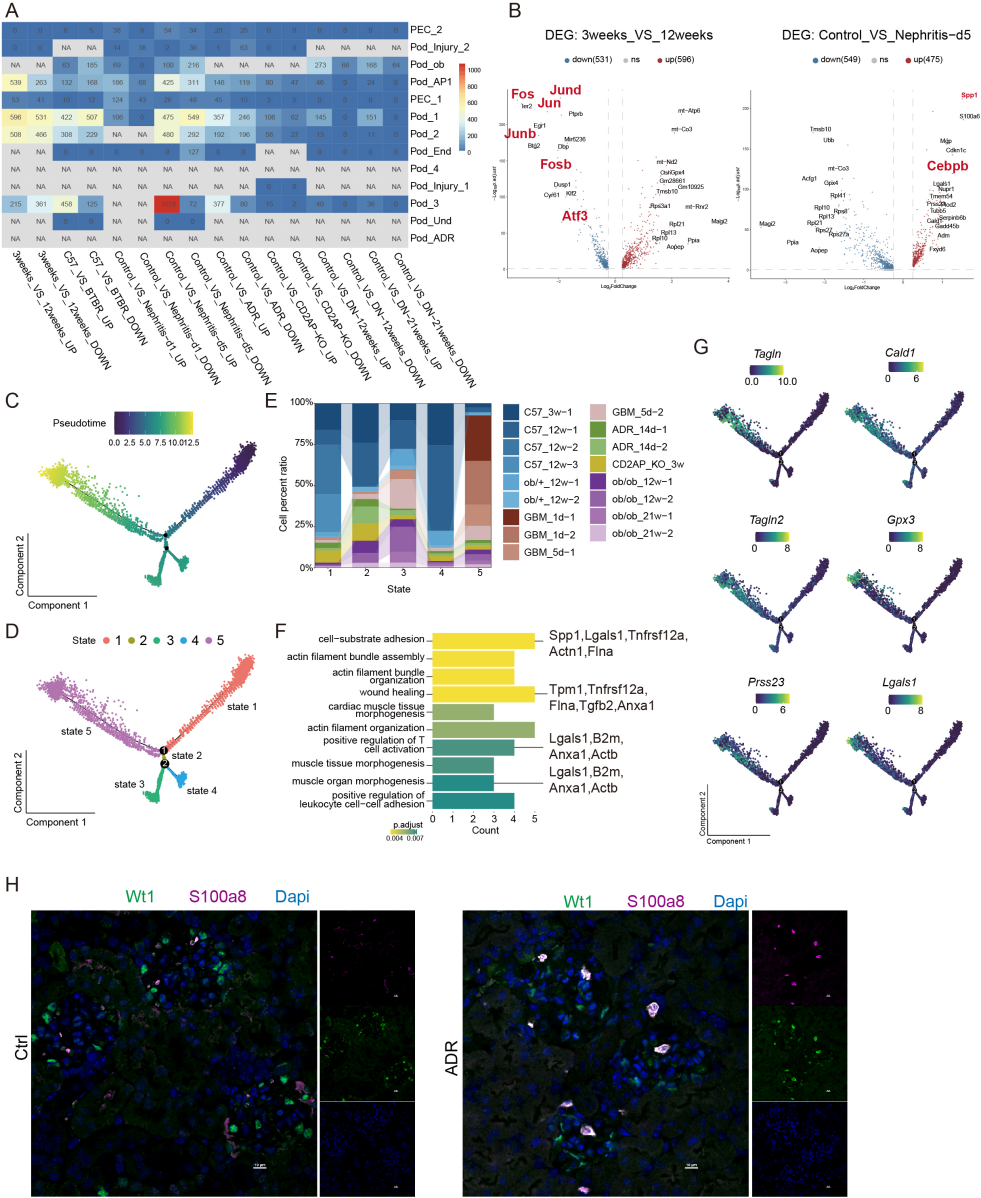
Shared molecular signature of podocyte injury across disease contexts. **(A)** Heatmap of the number of differentially expressed genes (DEGs) between different animal models and the control group. **(B)** Volcano plots of DEGs comparing different animal models to the control group (aging and nephritis). **(C)** Pseudotime trajectory analysis performed using monocle2, with different colors representing different pseudotime values. **(D)** Pseudotime trajectory analysis performed using monocle2, with different colors representing different cell states. **(E)** Proportion of podocytes from different animal models in each state of the pseudotime analysis. **(F)** Bar chart showing the results of GO enrichment analysis for state 5. **(G)** Expression patterns of key genes along the pseudotime axis in the pseudotime analysis. **(H)** Representative IF image of S100a8-positive podocytes in the healthy control (left) and ADR model (right) groups. Scale bar = 10 μm.

To identify common injury mechanisms, we compared the transcriptional profiles of podocyte subclusters between disease and control groups, quantifying differentially expressed genes (DEGs). A substantial number of DEGs were identified in POD_AP1 across different age groups and at the 5-day nephritis time point. Additionally, POD_1, POD_2, and POD_3 also exhibited numerous DEGs under conditions of aging, diabetes, and nephritis (**Figure 3A**). Notably, POD_1—a major constituent of the podocyte population—significantly downregulated AP-1-related molecules (*Fos*, *Junb*, *Jun*, *Fosb*, *Atf3*) with advancing age. In the nephritic state, *Spp1* and *Cebpb* was markedly upregulated (**Figure 3B**). Pseudotime analysis of podocyte subclusters distributed them across five distinct states, with a majority of injury-associated subclusters coalescing in State 5 (**Figures 3C–E**). GO analysis revealed that State 5 is primarily associated with cell–matrix interactions, actin filament assembly and organization, wound healing, morphogenesis of cardiac and muscle tissue, positive regulation of T-cell activation and positive regulation of leukocyte–cell adhesion (**Figure 3F**). Key injury-related genes, including *Tagln*, *Cald1*, *Tagln2*, *Gpx3*, *Press23*, and *Lgals1*, were also predominantly highly expressed in this state (**Figure 3G**). These characteristics suggest that State 5 likely represents a characteristic injury or repair state, involved in podocyte response, remodeling, and immunomodulatory processes following damage. Notably, the immunofluorescence staining of renal tissues confirmed the co-expression of Wt1 and S100a8 in ADR mice, demonstrating that some podocytes highly express S100a8 in this disease model (**Figure 3H**).

### 3.4 Molecular signature of mesangial cell injury with a focus on diabetic nephropathy

**Figure 4:**
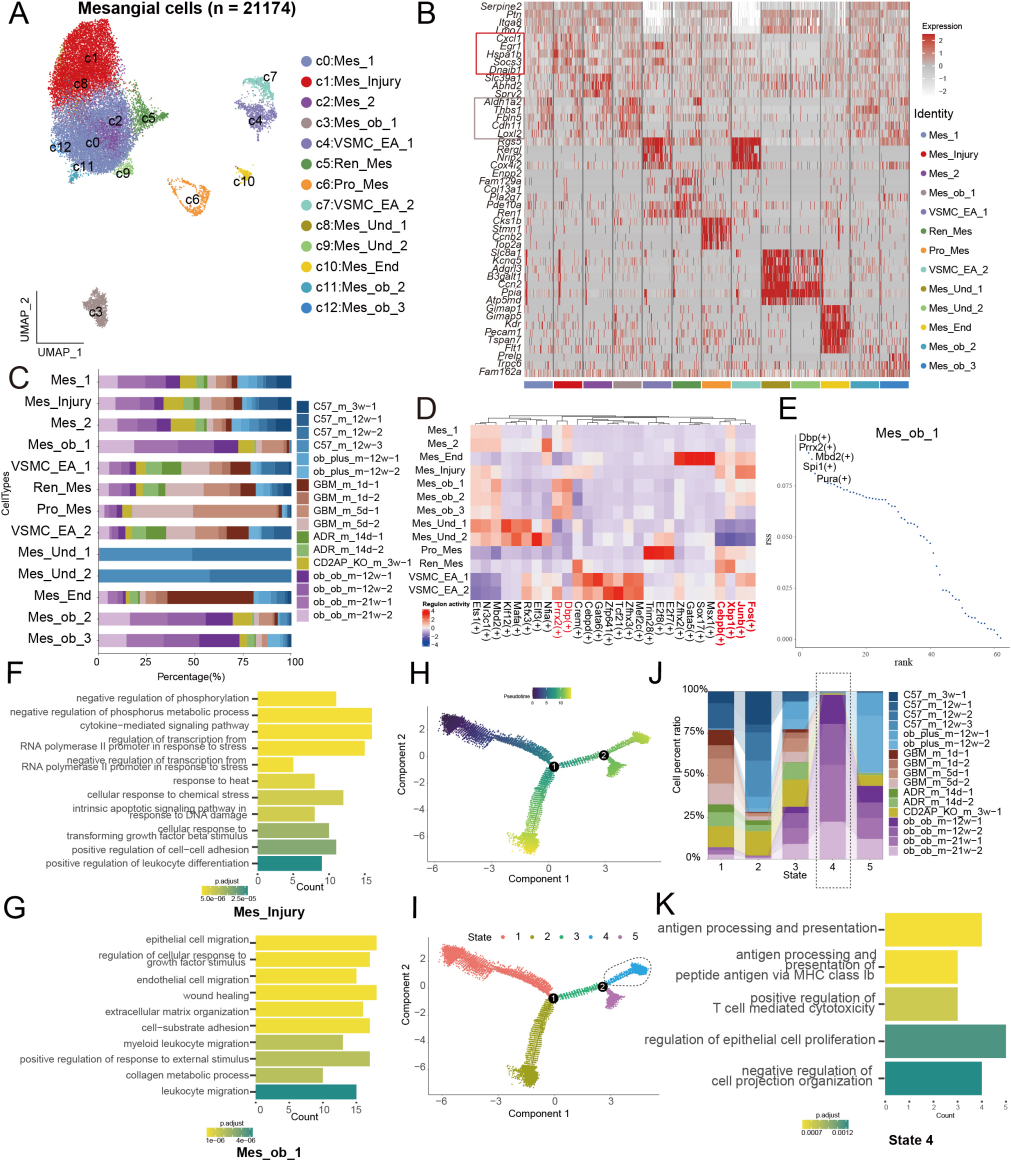
Molecular signature of mesangial cell injury with a focus on diabetic nephropathy **(A)** UMAP of 21,174 Mesangial cells. **(B)** Heatmap of expression of cell type-specific marker genes for each mesangial cell subset. The size of each dot indicates the percentage of cells expressing the marker gene, while the color intensity reflects the average expression level of the marker gene. **(C)** Bar chart of mesangial cell subset proportions across different animal models. **(D)** Heatmap of characteristic transcription factors in different mesangial cell subsets. **(E)** The characteristic transcription factors of Mes_ob_1. **(F, G)** Bar chart of GO enrichment analysis for different mesangial cell subsets. **(H)** Pseudotime trajectory analysis performed using monocle2, with different colors representing different pseudotime values. **(I)** Pseudotime trajectory analysis performed using monocle2, with different colors representing different cell states. **(J)** Proportion of podocytes from different animal models in each state of the pseudotime analysis. **(K)** Bar chart showing the results of GO enrichment analysis for state 4.

Mesangial cells (MCs) are crucial for maintaining glomerular structural integrity, regulating filtration rate, synthesizing or degrading extracellular matrix (ECM), and producing various cytokines and bioactive molecules (19). We performed reclustering analysis on MCs, identifying 13 distinct subclusters (**Figure 4A**), which were annotated based on their characteristic gene expression profiles (**Figure 4B**). Among these, we focused on cluster 1, annotated as Mes_Injury, which was predominantly derived from multiple disease groups (**Figure 4C**). This subpopulation highly expressed *Cxcl1*, *Egr1*, *Hspa1b*, *Socs3*, and *Dnaib1* (**Figure 4D**). Notably, we identified several disease-specific MC subclusters—clusters 3, 11, and 12—that were markedly expanded in *ob/ob* mice (**Figure 4C**). Specifically, Mes_ob_1 (cluster 3) exhibited a transcriptome distinct from other MCs and appeared as an outlier in UMAP visualization (**Figure 4A**). It was characterized by high expression of *Aldh1a2*, *Thbs1*, *Fbln5*, *Cdh11*, and *Loxl2* (**Figure 4B**). Transcription factor analysis revealed that Mes_Injury highly expressed *Fos*, *Junb*, *Xbp1*, and *Cebpb*, while all *ob/ob*-derived MCs shared high expression of *Dbp* and *Prrx2* (**Figure 4D**). Mes_ob_1 uniquely expressed *Mbd2*, *Spi1*, and *Pura* (**Figure 4E**). Of translational relevance, *Prrx2* is involved in development, tissue repair, and epithelial–mesenchymal transition (EMT), regulating ECM remodeling and cell migration (20, 21). *Spi1*, primarily expressed in the immune system, is a key regulator of hematopoiesis and immune responses (22, 23). Functionally, GO enrichment analysis indicated that Mes_Injury is primarily associated with intracellular signaling regulation and stress responses (**Figure 4F**), whereas Mes_ob_1 is more enriched in processes related to cell migration, tissue repair, and cell–matrix interactions (**Figure 4G**). Pseudotime trajectory analysis of all MC subclusters (**Figure 4H**) showed that *ob/ob*-derived MCs were concentrated in State 3 (**Figures 4I, J**), where enriched genes were functionally linked to antigen presentation, immune response, regulation of epithelial cell proliferation, and negative regulation of cell projection organization (**Figure 4K**).

### 3.5 A Shared glomerular endothelial cell injury subpopulation is induced across diverse disease models

**Figure 5:**
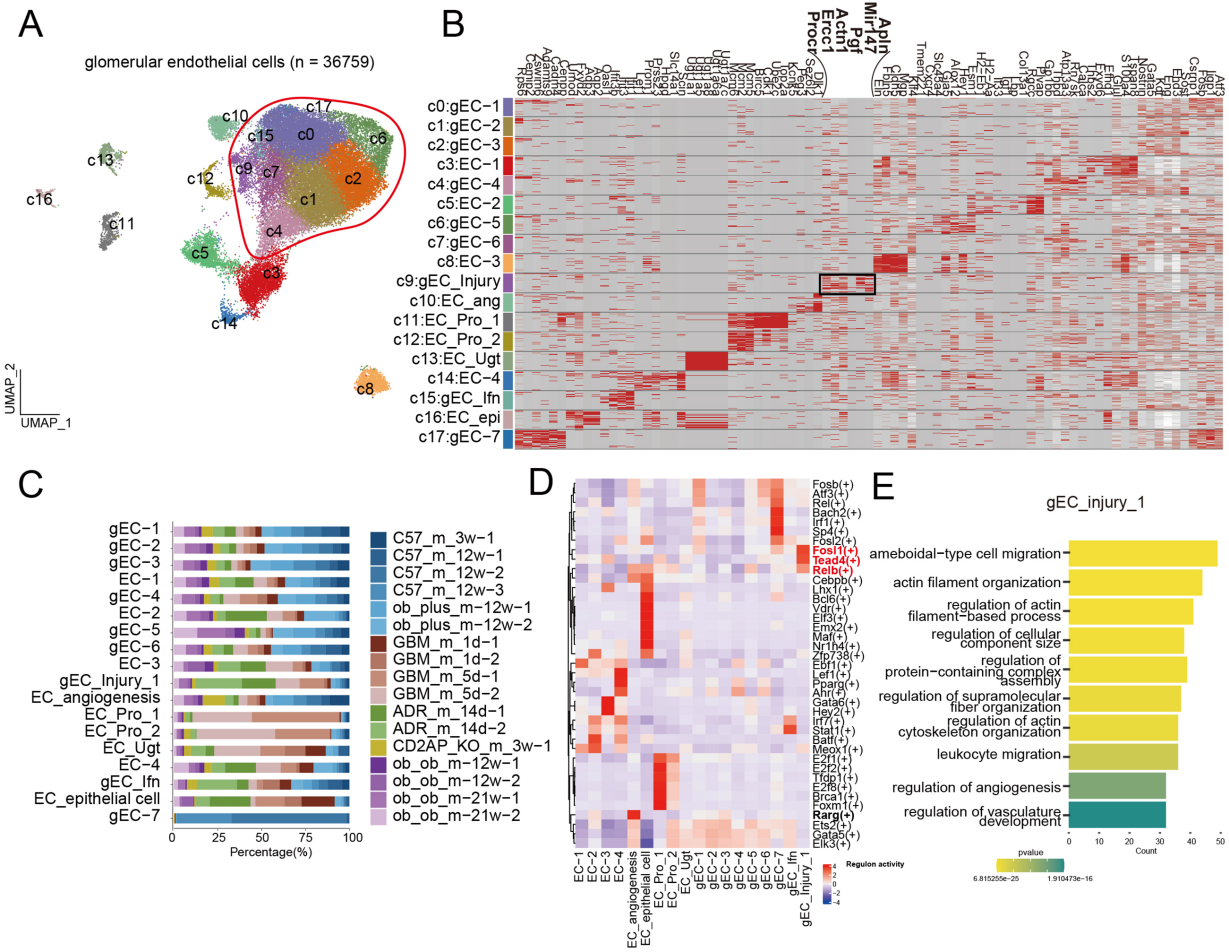
**(A)** shared glomerular endothelial cell injury subpopulation is induced across diverse disease models. **(A)** UMAP of 36,759 glomerular endothelial cells. **(B)** Heatmap of expression of cell type-specific marker genes for each glomerular endothelial cell subset. The size of each dot indicates the percentage of cells expressing the marker gene, while the color intensity reflects the average expression level of the marker gene. **(C)** Bar chart of glomerular endothelial cell subset proportions across different animal models. **(D)** Heatmap of characteristic transcription factors in different glomerular endothelial cell subsets. **(E)** The characteristic transcription factors of gEC_injury_1.

Glomerular endothelial cells (gECs) are essential for maintaining the integrity of the filtration barrier, regulating molecular exchange, balancing coagulation and anticoagulation, and providing structural support (9). Their injury contributes to increased permeability (leading to hematuria and proteinuria) and microthrombosis, thereby accelerating kidney disease progression (9). We performed reclustering of gECs, identifying 18 subclusters that included 9 distinct subpopulations with unique transcriptional profiles (**Figures 5A, B**). Notably, gEC heterogeneity was not primarily driven by specific disease contexts (**Figure 5A**). However, cluster 9 emerged as an exception, being predominantly derived from multiple disease groups (**Figure 5C**). We annotated cluster 9 as gEC_Injury, identifying it as a potential common injury-specific subpopulation (**Figure 5B**). The gEC_Injury subpopulation was characterized by high expression of *Apln*, *Mir147*, *Pgf*, *Actn1*, *Ercc* and *Procr* (**Figure 5B**). Key transcription factors defining this cluster included *Fosl*, *Tead4*, and *Relb* (**Figure 5D**), which are known regulators of cell proliferation, migration, immune response, and tissue repair (24–26). Consistent with this molecular signature, GO analysis indicated that gEC_Injury is functionally enriched in processes related to cell migration, cytoskeletal regulation, immune response, as well as angiogenesis and vascular development (**Figure 5E**).

### 3.6 Widespread alterations in glomerular endothelial cell subpopulations induced by diverse disease models

**Figure 6:**
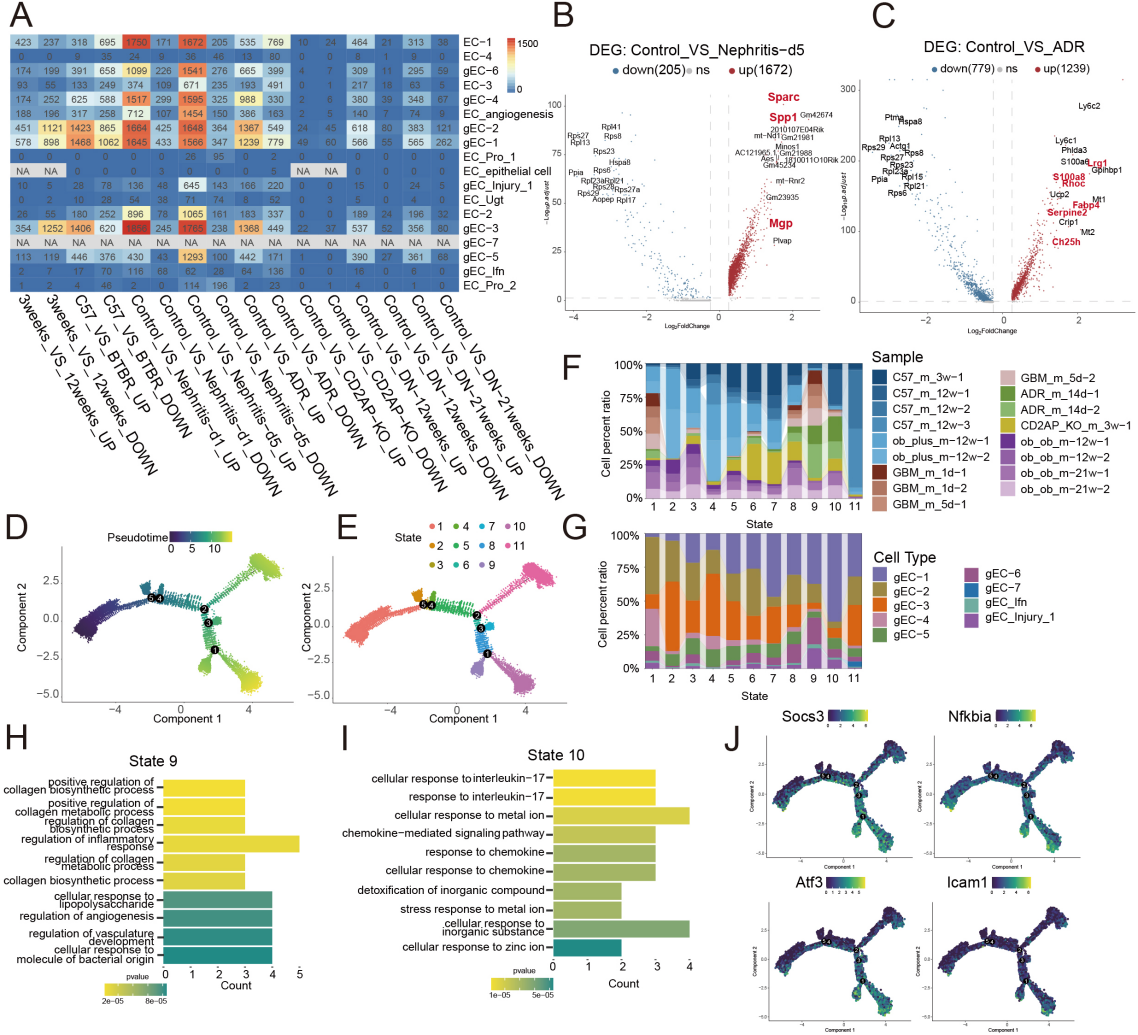
Widespread alterations in glomerular endothelial cell subpopulations induced by diverse disease models, **(A)** Heatmap of the number of differentially expressed genes (DEGs) between different animal models and the control group. **(B)** Volcano plots of DEGs comparing nephritis_d5 animal models to the control group. **(C)** Volcano plots of DEGs comparing ADR animal models to the control group. **(D)** Pseudotime trajectory analysis performed using monocle2, with different colors representing different pseudotime values. **(E)** Pseudotime trajectory analysis performed using monocle2, with different colors representing different cell states. **(F)** Proportion of podocytes from different animal models in each state of the pseudotime analysis. **(G)** Proportion of podocytes from glomerular endothelial cell subsets in each state of the pseudotime analysis. **(H)** Bar chart showing the results of GO enrichment analysis for state 9. **(I)** Bar chart showing the results of GO enrichment analysis for state 10. **(J)** Expression patterns of key genes along the pseudotime axis in the pseudotime analysis.

Transcriptomic analysis revealed substantial differential gene expression in gEC_1, gEC_2, gEC_3, gEC_4, and gEC_6 under nephritis and ADR conditions, with a predominant pattern of transcriptional upregulation (**Figure 6A**). Among these, gEC_1 and gEC_2 exhibited the most pronounced changes (**Figure 6A**). Disease-specific responses were observed for these two subpopulations: gEC_1 significantly upregulated *Spp1*, *Sparc*, and *Mgp* in the 5-day nephritis model (**Figure 6B**), whereas gEC_2 was characterized by elevated expression of *Lrg1*, *S100a8*, *Fabp4*, *Serpine2*, and *Ch25h* in the ADR model (**Figure 6C**). Pseudotime trajectory analysis (**Figures 6D–J**) further delineated injury-associated states. State 9 was predominantly populated by cells from disease groups and served as the primary distribution zone for the gEC_Injury subpopulation (**Figures 6E–G**). State 10 was also largely composed of disease-derived cells, with the ADR model being the major contributor (**Figures 6E–G**). Functional characterization showed that State 9 was significantly enriched for biological processes related to collagen synthesis and metabolism, inflammatory responses, angiogenesis, and responses to bacterium-derived molecules (**Figure 6H**). In contrast, State 10 was primarily associated with cellular responses to interleukin-17 and chemokines, chemokine-mediated signaling pathways, detoxification of inorganic compounds, and responses to metal ions and inorganic substances (**Figure 6I**). Furthermore, key genes involved in cellular stress, immune response, proliferation, and differentiation—including *Socs3*, *Nfkbia*, *Atf3*, and *Icam1*—were highly expressed in both State 9 and State 10 (**Figure 6J**).

### 3.7 Specific ligand–receptor interactions in injury-related cell subsets

**Figure 7:**
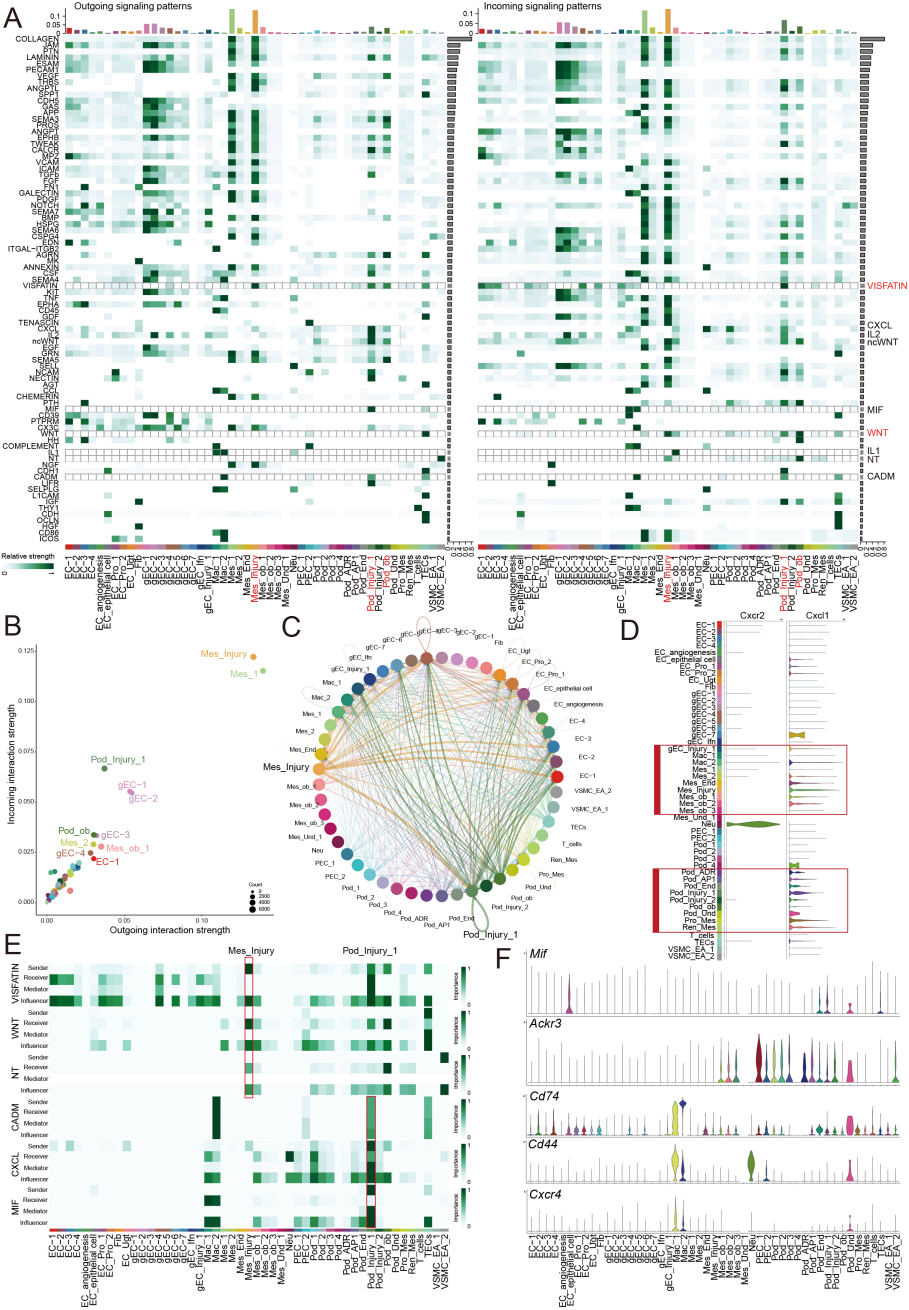
Specific ligand–receptor interactions in injury-related cell subsets. **(A)** Heat map of ligand–receptor interaction network between glomerular cell subsets and other cell types under disease conditions. **(B)** Quantification of ligand–receptor relationship prominence among glomerular cell subsets **(C)** Chord diagram showing VISFATIN-mediated ligand–receptor interactions between glomerular cell subsets and other cell subpopulations. **(D)** Violin plot displaying Cxcl1/CXCR2 expression levels in glomerular subsets and other cell subpopulations under injury conditions. **(E)** Heat map of CADM, CXCL, and MIF ligand–receptor pathways involved in macrophage and neutrophil recruitment, illustrating the receivers, mediators, and influencer among glomerular subsets and other cell subpopulations. **(F)** Violin plot showing the expression of Mif, Ackr3, Cd74, Cd44, and Cxcr4 in glomerular cell subsets and other cell types under injury conditions.

We analyzed the interactions between glomerular cell subsets and other cell types under disease conditions (**Figure 7A**) and found that mesangial cells exhibited particularly prominent ligand–receptor relationships with other cell subpopulations (**Figure 7B**). Notably, we observed that VISFATIN-mediated ligand–receptor interactions were primarily identified between Mes_injury and Pod_injury_1 subsets (**Figure 7C**). This suggested that extracellular NAMPT derived from mesangial cells may directly or indirectly bind to and activate Toll-like receptor 4 (TLR4) on podocytes. Elevated Visfatin levels are known to promote chronic inflammation via the TLR4/NF-κB pathway (27). Consistent with our findings (**Figure 7D**), prior studies have reported that mesangial cells may upregulate Cxcl1 expression upon injury (16). Furthermore, we detected high Cxcl1 expression in other injured podocyte subsets. This may represent a shared mechanism by which mesangial cells and podocytes collectively facilitate the infiltration of CXCR2-high neutrophils in glomerular diseases. Injured podocytes may also recruit macrophages and neutrophils via CADM, CXCL, and MIF pathways (**Figure 7E**). Among glomerular cell types, Mif was predominantly upregulated in Pod_injury_1 under injury conditions, while its receptors—Cd74, Cd44, and Cxcr4—were highly expressed in macrophages, neutrophils, and Pod_Und subpopulations (**Figure 7F**). Existing evidence supports a key role for MIF signaling in inflammatory recruitment (28). Recent studies suggest that MIF–CD44 interactions may contribute to parietal epithelial cell injury and the development of focal segmental glomerulosclerosis (1, 15, 29). Our results indicated that this may represent a broad mechanism underlying glomerular inflammatory recruitment following podocyte injury across multiple disease contexts.

## 4 Discussion

With an increasing number of kidney diseases found to have genetic factors, and the gradual identification of susceptibility genes for diseases such as FSGS, IgAN, and DN, there has been a strong interest in exploring the molecular mechanisms of kidney diseases (30–32). In-depth analysis of the transcriptional profile characteristics of glomerular cells under different pathological and physiological conditions at the molecular level is expected to completely transform traditional diagnostic strategies and open up new avenues for the treatment of kidney diseases. Therefore, we used single-cell RNA sequencing (scRNA-seq) to characterize the cells of the glomerulus and further identify characteristic changes in cell states under pathological conditions.Integrating insights from genomics, we discovered that susceptibility genes for different glomerulopathies are enriched in distinct cell types. Specifically, FSGS-associated genes are predominantly highly expressed in podocytes and mesangial cells, whereas DN-associated genes lack such cell type-specific enrichment. These findings suggest that the primary target—or the initially injured cell type—may differ significantly across various glomerular diseases.

To date, gene expression in the glomerulus has been examined almost exclusively in cell populations rather than at the single-cell level. Therefore, it is not yet clear whether there is cellular heterogeneity within the glomerular cells. To address this fundamental question, we performed re-clustering analysis on 11,637 podocytes, 21,174 glomerular endothelial cells, and 36,759 mesangial cells, systematically delineating the molecular alterations in glomerular cell subpopulations under disease conditions. Notably, we identified both shared and disease-specific molecular signatures across different pathological contexts. Within podocytes, a shared injury subpopulation emerged, characterized by its association with actin cytoskeleton organization and high expression of *Tagln*, *Cxcl1*, *Crct1*, *Angptl7*, *Tgfb2*, and *Flna*. Transgelin, encoded by *Tagln*, is an actin-binding protein that regulates actin polymerization, bundling, or cross-linking (33). Previous studies have shown that asparagine endopeptidase, Cyclosporine A, and the Smad3 inhibitor SIS3 can protect podocytes and reduce proteinuria by inhibiting or cleaving transgelin (34–36). Additionally, LPS and serum albumin (SA) significantly upregulate *CXCL1* expression in podocytes (37, 38). In contrast, disease-specific podocyte subpopulations were also observed: an ADR-induced cluster highly expressing *Selenbp1*, *Lpar1*, *S100a8*, *S100a9*, and *Sult1a1*, and an *ob/ob*-specific cluster enriched in *Endou*, *Cd200*, *Lgmn*, *Il18*, *Dmpk*, and *Spon2*. Podocytes are terminally differentiated epithelial cells. S100a8 typically forms a heterodimer with S100a9, known as calprotectin (39). To provide a solid cellular context for interpreting those findings, we focused validation on WT1-positive podocytes due to its specificity and conservation. The co-expression of S100a8 in WT1-positive cells directly demonstrates that these podocytes themselves are in an activated or injured state. The S100a8/S100a9 heterodimer can be released into the extracellular space, where it binds to receptors such as TLR4 and RAGE, activating neighboring podocytes, tubular epithelial cells, and infiltrating immune cells. The transcription factor (TF) activities also differed markedly among these subpopulations. For instance, injured podocytes exhibited activation of TFs such as *Bcl3*, *Rela*, *Fosl1*, and *Nfkb2*, providing deeper insight into the upstream regulatory events during podocyte injury. The AP-1 pathway appears to play a dual, context-dependent role in podocytes: while it is downregulated during maturation (as seen in 12-week-old versus 3-week-old mice (15)) and may support a differentiated state at low homeostatic levels, its sustained, high-level activation in the disease-associated POD-AP1 subpopulation is strongly linked to dedifferentiation and injury.

Under diabetic conditions, mesangial cells undergo distinct transcriptional reprogramming and give rise to a unique subpopulation characterized by high expression of *Aldh1a2*, *Thbs1*, *Fbln5*, *Cdh11*, and *Loxl2*. ALDH1A2, a rate-limiting enzyme in retinoic acid (RA) synthesis, is essential for kidney development (40) and catalyzes the oxidation of retinol to RA (41). Dysregulation of RA metabolism is considered a key pathogenic factor in diabetic nephropathy(DN) (42, 43). D-site-binding protein (DBP), encoded by *DBP*, regulates retinol metabolism and TGF-β1 signaling (44). Our data indicate that *DBP* is highly activated in this specific mesangial subpopulation in DN, suggesting that DBP may drive retinol metabolic dysregulation, as reflected by the elevated *Aldh1a2* expression. THBS1, encoded by *Thbs1*, interacts with multiple extracellular matrix (ECM) components—including collagen V and VII, fibrinogen, jagged1, laminin, MMP-2, MMP-9, TGF-β, CD36, and von Willebrand factor—all of which are closely associated with mesangial ECM homeostasis (45). *Thbs1* has been identified as a hub gene in DN mesangial cells (46). Furthermore, *Fbln5*, *CDH11*, and *Loxl2* are all strongly linked to ECM regulation and fibrotic processes (47–50). We also observed activation of the transcription factor *Prrx2* in DN mesangial cells, which may further reinforce TGF-β–mediated fibrotic signaling. Together, these findings indicate that mesangial cells in DN acquire a pronounced pro-fibrotic, matrix-secretory phenotype. In addition, consistent with previous reports, we confirmed that injured mesangial cells highly express *Cxcl1* (16). Pseudotime analysis further revealed that mesangial cells from ob/ob mice are enriched in antigen presentation-related functions. These results imply that in diseased states, mesangial cells may adopt immunomodulatory properties and engage in active crosstalk with immune cells.

In contrast to podocytes and mesangial cells, our analysis did not reveal disease-specific glomerular endothelial cell (gEC) subpopulations across different pathological models. Instead, gECs exhibited a common injury response, characterized by a shared subpopulation with elevated expression of *Apln*, *Mir147*, *Pgf*, *Actn1*, *Ercc1*, and *Procr*. Apelin, encoded by *Apln*, is an endogenous secreted peptide that binds to the orphan receptor APJ (APLNR) and participates in vasodilation, blood pressure regulation, cardiac contractility, and energy metabolism (51). ACTN1, an actin-crosslinking protein, serves as a key component of the cytoskeleton and contractile apparatus in cells (52). The upregulation of actin filament-related genes in endothelial cells often represents an adaptive response to mechanical, chemical, or inflammatory stimuli. This response may serve protective roles—such as reinforcing barrier function or promoting angiogenesis—but may also precede or drive pathological processes, including Endothelial-to-Mesenchymal Transition and vascular stiffening (52–55).

Our study has several limitations. First, our analysis was confined to four distinct disease models and healthy controls and did not include secondary glomerulopathies such as ANCA-Associated Glomerulonephritis. Incorporating a broader spectrum of disease models such as membranous nephropathy and different disease stages would strengthen the generalizability of our conclusions. Second, we focused exclusively on three major glomerular cell types—podocytes, mesangial cells, and endothelial cells—and did not explore other relevant populations such as immune cells, which are known to interact closely with glomerular cells. Third, our study lacks experimental functional validation of the key candidate genes identified. Direct evidence from gain- or loss-of-function experiments is needed to confirm their causal roles in disease pathogenesis, which remains an important objective for future research. Finally, the inclusion of a larger number of patient-derived samples will be essential to more comprehensively evaluate the consistency and translational relevance between mouse models and human disease. In summary, our study has generated a comprehensive and standardized scRNA-seq atlas of the murine glomerulus. We have demonstrated the key role of changes in cell composition in driving extensive expression differences, observed changes in cell type-specific gene expression, and identified patterns of injury under disease conditions. These findings provide important insights into kidney diseases and lay the foundation for future research aimed at understanding and treating kidney diseases.

## 5 Conclusions

The pathogenic mechanisms of glomerular diseases—both shared and unique across different types of glomerular diseases—remain inadequately understood. By systematically comparing four distinct disease models and healthy controls, we uncovered two distinct response patterns and one common response across major cell types, and elucidates the pathogenic role of a novel disease-specific cell subpopulation in diabetic nephropathy.

## Data Availability

The datasets presented in this study can be found in online repositories. The names of the repository/repositories and accession number(s) can be found in the article/**Supplementary Material**.

## Supplementary material

Supplementary Table 1 Data information. Information for disease and control groups includes samples details.

Supplementary Table 2–6 Cluster information. The comprehensive details for each subset generated from every clustering step, including quality, annotations, marker genes, and their top 50 signature genes.
